# How supernatural and scientific beliefs influence individual cancer prevention behaviors: an empirical study of young Chinese adults

**DOI:** 10.3389/fpubh.2025.1711669

**Published:** 2025-12-04

**Authors:** Qinliang Liu, Hepeng Jia

**Affiliations:** School of Communication, Soochow University, Suzhou, China

**Keywords:** cancer prevention, young adults, scientific attitudes, supernatural/religion, health control beliefs

## Abstract

**Introduction:**

The global increase in the incidence of early-onset cancer underscores the urgent need to understand the behavioral determinants of prevention effectiveness. This study aimed to examine the fundamental roles of supernatural and scientific beliefs in shaping health-related cognitions and their potentially conflicting influences on cancer prevention behaviors.

**Methods:**

Based on health locus of control (HLOC) theory, a dual-pathway attitudinal model was developed and tested using a two-step procedure to validate the model with data from a national survey (*N* = 938) of young Chinese adults.

**Results:**

The results indicated that both supernatural and scientific beliefs significantly predicted cancer control beliefs and prevention behaviors. Specifically, Chinese supernatural beliefs negatively predicted cancer prevention behaviors through cancer fatalism (external HLOC). In contrast, positive scientific attitudes were positively associated with cancer prevention behaviors through cancer controllability (internal HLOC). Furthermore, positive scientific attitudes mitigated the detrimental effects of supernatural beliefs on cancer prevention behaviors by reducing cancer fatalism and strengthening perceived cancer controllability.

**Discussion:**

These findings elucidate the underlying factors contributing to both effective and ineffective cancer prevention and identify potential pathways for intervention and education. Both theoretical and practical implications are discussed.

## Introduction

Cancer remains the leading cause of death globally, contributing to a growing disease burden (1, 2). While historically concentrated in older populations, significant increases in cancer incidence across all adolescent and young adult age groups (15–39 years) in recent decades indicate an emerging early-onset cancer trend (3, 4). Notably, the incidence of obesity-related cancers (e.g., multiple myeloma, colorectal, uterine corpus, gallbladder, kidney, and pancreatic cancers) is rising rapidly among young adults (5, 6), underscoring the critical importance of early detection and prevention for this demographic.

Despite significant advancements in cancer treatment (7), effective primary prevention remains crucial, with approximately 40% of cancer cases being preventable.^1^ Consequently, promoting cancer prevention represents one of the most significant public health challenges of the 21st century (8). Its efficacy is hindered by multifaceted factors, including socioeconomic status, health knowledge, and healthcare access (9), which complicate intervention efforts. Crucially, socioeconomic factors alone cannot fully explain disparities in cancer prevention outcomes (10, 11); cultural, cognitive, and behavioral factors also play integral roles (12). Research has increasingly focused on cognitive determinants—such as attitudes, beliefs, and values—that influence success or failure in cancer-related health behaviors (13, 14).

Among these, supernatural/religious and scientific beliefs constitute fundamental cognitive frameworks that shape distinct cancer control beliefs. Adherence to these worldviews influences diverse behaviors, including health-related actions (15). Prior research indicates that supernatural/religious beliefs often externalize health control perceptions (16) and are associated with pessimistic attitudes (e.g., fatalism) and adverse health outcomes (17, 18). Conversely, scientific literacy fosters self-efficacy and health competence, promoting positive health expectations (19, 20). Therefore, these contrasting belief systems may critically predict cancer prevention behaviors.

Popular discourse frequently positions supernatural/religious beliefs in opposition to science, framing them as conflicting truth claims about the world (21). This “conflict thesis” posits inherent incompatibility, suggesting that scientific advancement diminishes religious influence (22)—a paradigm that shaped early empirical research. Extensive studies have examined their relative effects on attitudes toward public issues such as climate change (23), vaccination (24, 25), and biotechnologies (26–29). However, most research originates from Western, predominantly Christian contexts, neglecting non-institutionalized supernatural traditions such as Chinese Daoist and Confucian beliefs. This study aimed to address this gap by examining whether supernatural beliefs and scientific attitudes conflict in influencing health behaviors within China’s unique cultural context.

Furthermore, the mechanisms through which these beliefs impact behaviors remain underexplored. The health locus of control (HLOC) framework provides a vital lens for examining cancer prevention pathways, as it helps explain why individuals succeed or fail in adopting health behaviors. Generally, individuals who perceive their health as determined by their own behaviors and actions are classified as having an internal HLOC, whereas those who attribute health outcomes to external factors—such as luck, chance, fate, God, or powerful others—are considered to have an external HLOC (30). Moreover, these differing health control beliefs are linked to distinct health outcomes. Research indicates that a stronger internal HLOC is associated with more positive health outcomes (31, 32), while an external HLOC tends to correlate with adverse health conditions (33). Grounded in this theoretical framework, this study employs a dual-pathway model to examine how reliance on supernatural beliefs and science shapes cancer control beliefs and prevention behaviors among young adults in China.

## Literature review

### Supernatural beliefs, cancer fatalism, and health prevention

Supernatural or religious beliefs are characterized by faith in a deity exercising control over human life (34). They form part of a cultural “tool kit” that individuals draw upon to interpret their symbolic and conceptual worlds (35). Basically, supernatural or religious beliefs function as a meaning-making system (36), shaping beliefs about the world and the self while explaining both mundane and extraordinary events (37). Supernatural/religious beliefs typically exert a strong influence on external health control beliefs and are consistently associated with cancer fatalism (38). Substantial research has explored the relationship between supernatural or religious beliefs and cancer fatalism (39, 40), revealing variations that depend on the type and level of religiosity (41, 42).

Cancer fatalism represents an outlook that attributes health events to external forces (e.g., God, divine power, supernatural agency), fostering a sense of powerlessness in preventing or surviving cancer (39). It is recognized as a critical barrier to cancer prevention (43). Extensive research demonstrates negative associations between fatalistic beliefs and cancer prevention behaviors across the cancer continuum—from information seeking (44, 45) and diagnosis (46) to screening (47, 48) and prevention (49). The prevailing explanation is that fatalism fosters passive resignation, deeming personal health efforts futile or unnecessary given perceived divine control over health outcomes (16). Consequently, individuals endorsing fatalistic beliefs exhibit reduced motivation for prevention behaviors, contributing to poorer health outcomes.

While research on supernatural or religious beliefs, cancer fatalism, and prevention has proliferated over the past two decades, studies remain concentrated in Western European, Latino, American, and African contexts, leaving China significantly underrepresented. Chinese supernatural/religious traditions—such as Buddhism, Taoism, and Confucianism—originate from East Asian cultural contexts and incorporate folk beliefs, including fortune-telling, feng shui, the five elements, and concepts of Tian (Heaven) and Di (Earth) (50). These supernatural beliefs (non-official religions) are widespread in China, substantially outnumbering adherence to institutional world religions such as Christianity, Judaism, or Islam (51). Crucially, a shared fatalistic belief in *ming* (fate)—interpreted as an uncontrollable force determining destiny—is deeply embedded within Chinese supernatural frameworks (52). For instance, Chinese individuals diagnosed with cancer often exhibit passive cognitive appraisals integrating the concept of *ming*, acknowledging supernatural powers beyond their control (53). Given religiosity’s cultural specificity (54) and China’s distinct socio-religious landscape, interactions between cultural attribution and fatalistic beliefs regarding cancer prevention behaviors may be particularly salient. Therefore, this study examines associations among Chinese supernatural beliefs, cancer fatalism, and cancer prevention behaviors in young Chinese adults.

### Positive science attitudes, cancer controllability, and cancer prevention

Science is knowledge established through observation and experimentation via an objective process and tries to disentangle helpful knowledge about the matter so that this knowledge can be put to practical use (55). Due to the division of labor in our societies, we must rely on scientists’ knowledge when using science-based technologies and making personal or civic decisions on issues that involve scientific knowledge (56). Thereby, positive attitudes toward and trust in science and scientists are required for the functioning of modern society. It can be regarded as an epistemological belief (worldview) that involves one’s core views about the forms and sources of knowledge and is expected to be related to acceptance of knowledgeable authority and reduced uncertainty (57). Furthermore, within the theoretical framework of trust in science, positive attitudes toward science are associated with an overall increase in trust in science (56), particularly among individuals who believe that scientific knowledge is objective and that knowledge derives from authority (57). Therefore, positive attitudes toward science may serve as a means of reducing uncertainty, fostering a perception of control over events and behavioral outcomes.

Attitudes toward science are a vital predictor of disease prevention. Laypeople depend on scientific experts’ knowledge when forming their views on science-based issues and making decisions about disease treatment (58). Individuals with a better understanding of science generally have less epistemically questionable beliefs and, consequently, tend to behave more in line with evidence-based guidelines (59). In addition, accumulated scientific knowledge also fosters health literacy and self-efficacy, which greatly contribute to the development of internal HLOC beliefs, closely related to disease prevention (19, 20).

Controllability—the antithesis of fatalism—emerges from an internal locus of control. It is defined as the belief in one’s capacity to determine internal states, behaviors, and environmental influences to achieve desired outcomes (60). Cancer controllability is a robust protective factor for cancer health, consistently associated with positive cancer prevention behaviors, such as improved diet and increased physical activity (61, 62). This occurs because individuals with high cancer controllability perceive their actions as directly impacting health outcomes, prompting deliberate engagement in health-promoting behaviors (30). Conversely, low cancer controllability correlates with risk behaviors characterized by poor impulse control, sensation-seeking, and disregard for adverse health consequences (63). Within cancer prevention, greater perceived cancer controllability thus predicts proactive health behaviors. Collectively, this demonstrates a clear relationship between positive science attitudes, enhanced cancer controllability, and cancer prevention behaviors.

### The present study

**Figure 1:**
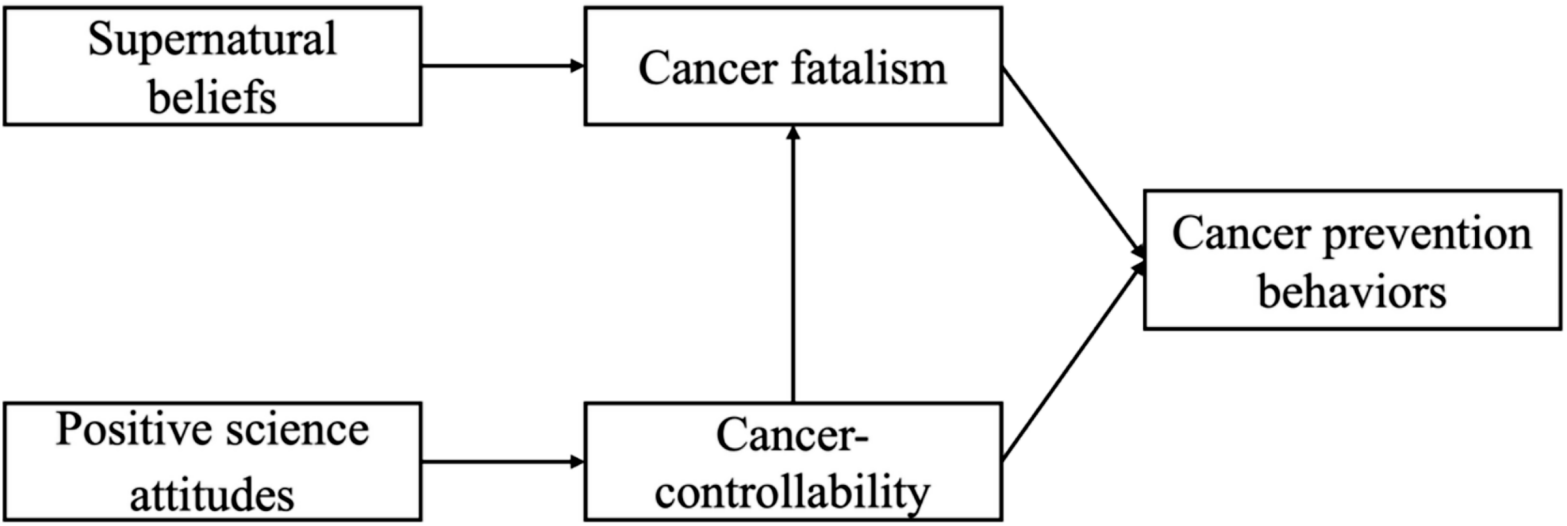
The framework of the research questions.

Although a growing body of research has reported that science and religion are significant predictors of health control beliefs and may be logically incompatible (64–66), relatively few empirical studies have investigated their tension in influencing individual health behaviors. This study aimed to examine how attitudes toward science and supernatural beliefs differ in predicting cancer prevention within a single model construct. With HLOC as the theoretical framework, we examined three research questions: (1) Do Chinese supernatural beliefs negatively predict cancer prevention behaviors through cancer fatalism? (2) Do positive attitudes toward science positively predict cancer prevention behaviors through cancer controllability? (3) Are Chinese religious beliefs and positive attitudes toward science competing predictors of cancer prevention behaviors? All the research questions are presented in Figure 1.

## Methods

### Participants and procedure

To answer the research questions, we conducted an online survey in China using the third-party survey company Credamo,^2^ which offers a national opt-in online panel of more than 1.5 million adults living in China. Participants aged 18 to 40 years were selected as the target sample, and a structured questionnaire was developed and pretested for this study.^3^ After providing their consent, participants were randomly selected to complete the questions regarding the study’s variables and demographic information. Data collection continued for 2 weeks, from 10 June to 24 June 2023. Overall, 1,002 people participated in the survey after excluding invalid responses (e.g., questionnaires completed in too short a time or incomplete questionnaires). Ultimately, 938 cases were retained in our study.^4^

Among the 938 participants, 606 (64.4%) were female, and the mean age was 24.91 years (SD = 4.83). The majority of the participants held a bachelor’s degree (approximately 70%) and reported a monthly income between US$400 and US$1300, and 79.1% identified as non-religious. The sample’s demographic characteristic is in line with the reality that young Chinese individuals who often engage in supernatural activities (e.g., praying in a temple) are typically highly educated female individuals. Therefore, this sample is reasonably representative (67).

### Measures

*Chinese supernatural beliefs. A self-administered scale was used to assess* Chinese supernatural beliefs with seven items. As Chinese supernatural *beliefs are considered* a non-official religion and there is no existing religious doctrine as a reference, the items were adapted from previous studies on Chinese religion or folk religion (68–70), and an additional 12 items were specifically generated as alternative metrics. Then, we invited 10 young adults to list the most frequently encountered folk *religious belief* items. After consulting with religion study expertise, a final version scale with seven items was adopted in this study [e.g., “A supreme heaven-T’ien (God in a Western context) in the universe,” “After a person passes away, their spirit and soul still exist,” “Divination and fortune-telling can predict the future”]. The respondents were asked to indicate their agreement with each statement on a 5-point Likert scale, ranging from *“1 = strongly disagree”* to “*5* = *strongly agree.”* We averaged the seven items to form *a supernatural beliefs* scale (*α* = 0.906, *M* = 3.48, *SD* = 0.85).

*Cancer fatalism*. Cancer fatalism was assessed using three items adopted from a previous study (71): “It seems that everything causes cancer,” “There is not much I can do to lower my chances of getting cancer,” and “There are so many different recommendations about preventing cancer, I do not know which ones to follow.” The responses were measured on a 5-point Likert scale, ranging from *“1 = strongly disagree”* to “5 = *strongly agree.”* We averaged the three items to create a cancer fatalism scale (*α* = 0.794, *M* = 2.73, *SD* = 0.87).

*Positive science attitudes*. We measured the participants’ positive science attitudes using five items from Wintterlin et al. (56), who validated the scale *and demonstrated its* good reliability and validity (Cronbach α = 0.70). The items were as follows: “Science and research can solve any problem,” “Science and research improve our lives,” “The benefits of science and research are greater than potential damage,” “Science should be allowed to explore everything without restriction,” and “One day, science will give us a complete picture of how nature and the universe work.” The participants responded to these questions on a 5-point Likert-type scale (1 = “strongly disagree” to 5 = “strongly agree”). We averaged the responses to the five items to create a positive science attitudes scale (*α* = 0.816, M = 3.72, SD = 0.50).

*Cancer controllability*. Cancer controllability was measured using a scale adapted from the World Assumption Scale (72). A total of four items were used to assess cancer controllability: “People’s misfortunes (cancer) result from mistakes they have made,” “Through our actions, we could prevent cancer from happening to us,” “If people took preventive actions, cancer could be avoided,” and “When bad things (cancer) happen, it is because people have not taken necessary actions to protect themselves.” The participants responded to these questions on a 5-point Likert-type scale (1 = “strongly disagree” to 5 = “strongly agree”). We averaged the responses to the *four* items to *create a cancer controllability scale* (*α* = 0.837, M = 3.50, SD = 0.76).

*Cancer prevention behaviors*. A set of four questions was utilized to assess the participants’ cancer prevention behaviors. The items were adopted from the annual Health Information National Trends Survey (HINTS) that was conducted by the US National Cancer Institute (NCI) and have been used by numerous researchers (43, 52). The items included the following: (a) regular exercise, (b) smoking avoidance, (c) fruit and vegetable intake, and (d) regular cancer check-ups. All participants were asked to indicate the extent to which they agreed with the listed questions on a 5-point scale (1 = “strongly disagree” to 5 = “strongly agree”). We averaged the responses to all four items to create a scale of cancer prevention behaviors (*α* = 0.76, M = 3.74, SD = 0.83).

### Analysis strategy

Structural equation modeling (SEM) with maximum likelihood estimation was used to test the overall model, and Pearson correlation coefficients were first used to assess the relationship between the studies’ variables. Afterward, a two-step procedure with the measurement model and the structural model was conducted to achieve the study’s aim. A measurement model was constructed to examine whether the observed variables provided a reliable reflection of the latent variables. We employed a structural model to examine the hypothesized effect pathways. Both models were evaluated using selected goodness-of-fit indices. When they fit most of the indices, they were regarded as acceptable.

The following indices were utilized to evaluate the goodness of fit of the model: The chi-square statistic (*χ*^2^), *χ*^2^/df, the standardized root mean squared residual (SRMR), the Root Mean Square Error of Approximation (RMSEA), the comparative fit index (CFI), the normed fit index (NFI), and the goodness of fit index(GFI). In this study, the model was considered to have a good fit if all path coefficients were significant at the level of 0.05, *χ*^2^/df was below 5, the SRMR was below 0.08, the RMSEA was below 0.08, and the CFI, GFI, and NFI were 0.90 or higher (73). IBM SPSS Statistics version 24.0 and AMOS 25.0 (IBM Corp., Armonk, NY) were used for data analyses. A probability level of *p* < 0.05 was used as the base level of statistical significance.

## Results

### Measurement model

For the measurement model, a confirmatory factor analysis was conducted on the correlation matrix of the observed variables. The initial measurement model demonstrated a good fit to the data: *χ*^2^(325): 1015.801, *p* < 0.001, *χ*^2^/df = 3.833, SRMR = 0.040, RMSEA = 0.055, GFI = 0.913, NFI = 0.898, and CFI = 0.922. However, when examining the factor scores for the items that referred to the latent construct “positive science attitude,” the item “science should be allowed to explore everything without restriction” showed a low factor loading of 0.041. Science should be allowed to explore everything without restriction” is factor of positive science attitudes, since its factor loading is less than 0.5, so this item was omitted from the construct of positive science attitudes. Finally, the adjusted measurement model showed an excellent fit: *χ*^2^ (276) = 731.541, *p* < 0.001, *χ*^2^/df = 3.325, SRMR = 0.040, RMSEA = 0.050, GFI = 934, NFI = 0.924, and CFI = 0.946.

To examine convergent validity, construct reliability (CR) and the average variance extracted (AVE) for the latent variables were analyzed. We have rewritten this section to make the expression clearer: To assess the measurement model, both convergent and discriminant validity were examined following commonly accepted criteria. Convergent validity (the measure items that should be under the same factor/variable do fall on the same factor) was evaluated using the Average Variance Extracted (AVE) and Composite Reliability (CR). According to the reference of AVE values exceeded 0.50 and CR values were above 0.70, the results in Table 1 indicating a good convergent validity. This suggests that the observed indicators effectively represent their corresponding latent constructs. Discriminant validity was tested using the Fornell–Larcker criterion. Specifically, the square root of each construct’s AVE (shown in bold on the diagonal of Table 1) was greater than its correlations with other constructs, demonstrating sufficient discriminant validity. Hence, each latent variable was empirically distinct from the others and measured unique conceptual dimensions. As shown in Table 1, the measurement model showed good convergent and discriminant validity, allowing for the subsequent analysis of the structural model.

**Table 1:** Parameters and indicators of convergent and discriminant validity.

Constructs	PSA	RB	SC	CF	CPB	AVE	CR
PSA	**0.757**					0.524	0.815
RB	−0.035	**0.750**				0.573	0.904
CC	0.220^***^	−0.036	**0.724**			0.594	0.853
CF	−0.183^***^	0.206^***^	−0.309^***^	**0.770**		0.563	0.794
CPB	0.256^***^	−0.001	0.311^***^	−0.465^***^	**0.729**	0.531	0.850

RB, religious beliefs; CF, cancer fatalism; PSA, positive science attitude; CC, cancer controllability; CPB, cancer prevention behaviors.

*** *p* < 0.001. The bold values on the diagonal represent the square root of the AVE for each construct.

### Construct model

**Figure 2:**
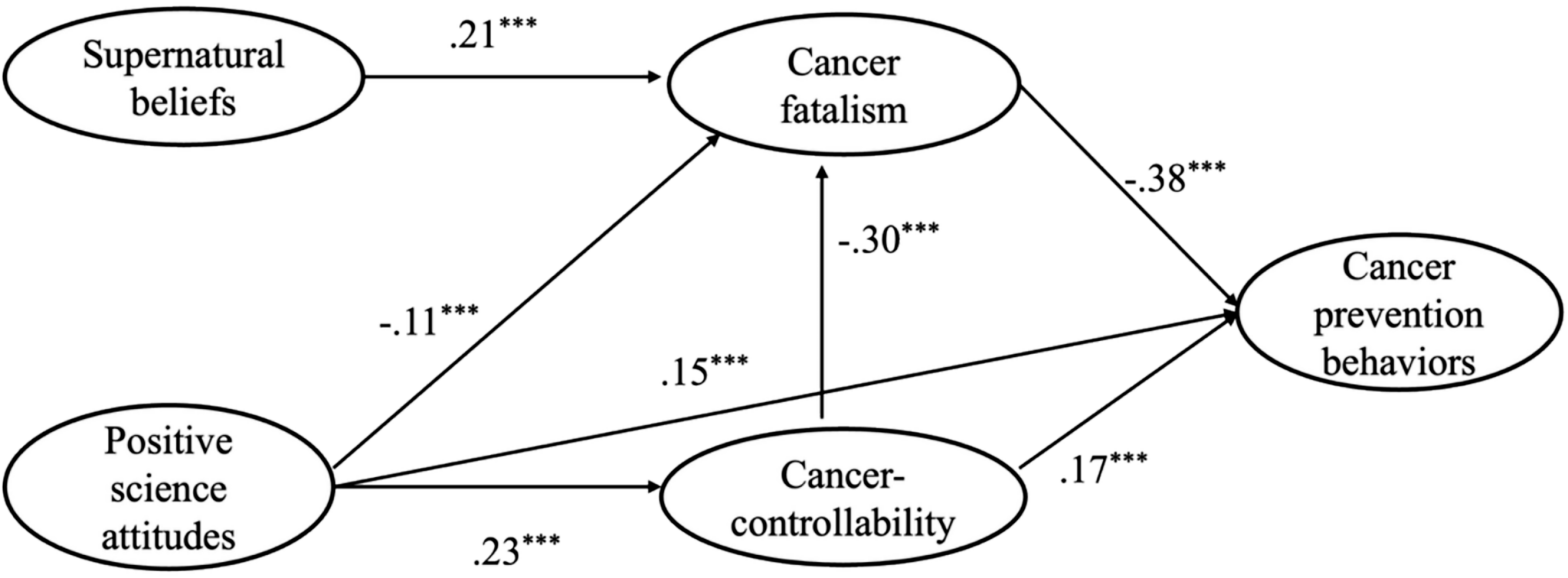
The path coefficient of the structural model.

Figure 2 presents the results of the structural model. The overall fit of the structural model was not better: *χ*^2^ (276) = 833.331 *χ*^2^/df = 3.704, *p* < 0.001, GFI = 0.926, CFI = 0.935, NFI = 0.914, SRMR = 0.056, and RMSEA = 0.054. According to modification indices (MI), we added the path from “positive science attitude” to “cancer prevention behaviors,” the path from “positive science attitude” to “cancer fatalism,” and the path from “cancer controllability” to “cancer fatalism.” The adjusted structural model showed a good fit: *χ*^2^ (276) = 589.358, *χ*^2^/df = 2.667, *p* < 0.001, GFI = 0.946, CFI = 0.961, NFI = 0.939, SRMR = 0.046, and RMSEA = 0.0423. The results of the pathway analyses for all hypotheses are presented in Figure 2.

As for the direct path coefficient, supernatural beliefs were positively associated with cancer fatalism (*β* = 0.21, *p* < 0.001), cancer fatalism was negatively associated with cancer prevention behaviors (*β* = −0.38, *p* < 0.001), positive science attitudes were positively associated with cancer controllability (*β* = 0.23, *p* < 0.001), and cancer controllability was positively associated with cancer prevention behaviors (*β* = 0.17, *p* < 0.001). In addition, the added path from positive science attitudes to cancer prevention behaviors (*β* = 0.15, *p* < 0.001), the path from positive science attitudes to cancer fatalism (*β* = −0.11, *p* < 0.001), and the path from cancer controllability to cancer fatalism (*β* = −0.30, *p* < 0.001) were also significant.

For the examination of indirect paths, the mediating effects were tested for significance using a bootstrap estimation procedure (with a bootstrap sample of 5,000), and the bootstrap test relied on the 95% confidence interval (CI). When the parameters of confidence interval between the lower limit (LLCI) and the upper limit (ULCI) includes zero, it indicates that the mediating effect is not significant. As shown in Table 2, all mediating effects were significant. Therefore, all research questions were answered.

**Table 2:** Standardized indirect effects.

Constructs	Estimate	SE	LLCI	ULCI
SB → CF → CPB	−0.08	0.01	−0.11	−0.05
PSA → CF → CPB	0.04	0.02	0.01	0.07
SC → CFB → CPB	0.04	0.01	0.02	0.07
PSA → SC → CF → CPB	0.03	0.01	0.01	0.04
Total indirect effect	0.03	0.03	−0.02	0.08
Total effect	0.17	0.54	0.09	0.30

SB, supernatural beliefs; CF, cancer fatalism; PSA, positive science attitude; SC, cancer controllability; CPB, cancer prevention behaviors; SE, standard error; LLCI, lower limit confidence interval, ULCI, upper limit confidence interval.

## Discussion

The global burden of cancer continues to grow, underscoring the critical importance of effective prevention. This study investigated whether supernatural/religious and scientific beliefs could be cognitive predictors of cancer prevention beliefs and behaviors. The results confirmed that both types of beliefs significantly predicted cancer-related control beliefs and behaviors, illustrating how social-cognitive resources shape health outcomes. Furthermore, drawing on HLOC theory, we established and demonstrated cancer fatalism (reflecting external HLOC) and cancer controllability (reflecting internal HLOC) as mediators through which these distinct worldviews influence cancer prevention behaviors.

Cancer prevention is influenced by a multitude of factors. Within the prevailing health communication paradigm, researchers have often sought to promote cancer prevention by using fear appeals and enhancing self-efficacy through various persuasive strategies (74–76). This study offers an alternative perspective for understanding individuals’ diverse beliefs and responses to cancer prevention. The findings indicated that supernatural beliefs function as antecedents to external HLOC (cancer fatalism) and are linked to cancer prevention behaviors, suggesting that sociocultural values play a significant role in shaping individuals’ health control beliefs and behaviors. This observation aligns with existing Western research, highlighting religion as a powerful determinant of fatalism—a major barrier to cancer prevention (17, 77). Specifically, the results suggested that supernatural beliefs function similarly to religion in fostering cancer fatalism and reducing prevention behaviors, offering a culture-specific explanation for the failure of cancer control initiatives in non-religious societies such as China. In addition, positive attitudes toward science demonstrated substantial potential in reinforcing cancer control beliefs and promoting cancer prevention behaviors.

This study addresses the “conflict thesis” concerning the relationship between supernatural beliefs and scientific attitudes. The results showed that supernatural beliefs indirectly and negatively predicted cancer prevention behaviors, whereas positive science attitudes indirectly and positively predicted such behaviors. In other words, both supernatural beliefs and scientific attitudes influenced young Chinese adults’ cancer prevention behaviors, but they operated in fundamentally opposing directions. Further supporting the conflict thesis, the interaction between beliefs derived from these two worldviews revealed that cancer controllability negatively predicted cancer fatalism. Moreover, positive science attitudes directly and indirectly—via cancer controllability—predicted cancer fatalism, reflecting underlying tensions between the two belief systems. However, contrary to a straightforward conflict model, positive science attitudes and supernatural beliefs did not exhibit conflict at the root level (as illustrated in the left part of Figure 2), but rather through their derived beliefs. This nuanced finding may be attributed to the distinctive characteristics of Chinese culture, in which both supernatural and scientific perspectives are inherently pragmatic, oriented primarily toward personal well-being and moral cultivation rather than toward describing reality or pursuing propositional truth (78). As a result, they function as independent cognitive systems with no ontological overlap or inherent conflict (79).

The findings regarding the “conflict thesis” in this study offer a nuanced perspective on how two distinct worldviews competitively influence cancer control beliefs and prevention behaviors among young Chinese adults. Positive science attitudes were found to counteract the detrimental effects of supernatural beliefs on cancer prevention through a dual-pathway mechanism—either directly reducing fatalistic perceptions or enhancing personal controllability. Furthermore, the calculation of cumulative effect coefficients revealed that the beneficial influence of scientific attitudes outweighs the adverse impact of supernatural beliefs, thereby collectively facilitating cancer prevention. However, this finding may be attributable to the characteristics of our sample—young adults with relatively high levels of education and scientific literacy, among whom a scientific worldview is likely predominant. This stands in contrast to studies conducted among older, less educated, or rural populations, which report higher levels of fatalism and a tendency to view cancer as “bad luck” or “divine punishment,” resulting in more passive cancer prevention behaviors (80–82). Such disparities suggest that individual engagement in cancer prevention reflects the relative emphasis placed on supernatural/religious versus scientific frameworks across different population groups. Overall, these findings elucidate the pathways through which scientific beliefs compete with supernatural beliefs in the context of a specific health issue, advancing our understanding of how divergent worldviews influence health-related control beliefs and behaviors.

Finally, these findings offer important implications for cancer prevention among young Chinese adults by identifying underlying cognitive factors and pathways that influence cancer prevention outcomes. Specifically, Chinese supernatural beliefs were closely associated with cancer fatalism, a key barrier to prevention, while positive science attitudes—linked to self-controllability—were found to mitigate fatalism and promote cancer prevention behaviors. This suggests that intervention efforts could adopt a dual strategy: reducing exposure to or influence of supernatural beliefs while simultaneously enhancing scientific literacy and internal self-control beliefs. Shifting cognitive perceptions of cancer controllability may represent a crucial approach to improving prevention, as effective health persuasion should be grounded in the epistemic belief that cancer can be controlled through personal action. Certainly, this proposition warrants further empirical investigation in future studies.

### Limitations and future research

Although this study makes several substantial contributions, some limitations should be acknowledged. First, the use of a self-developed scale to measure supernatural beliefs may have affected the validity of the construct and the robustness of the findings. Future research is encouraged to adopt well-validated instruments, such as the one developed by Tratner et al. (83), to enhance measurement reliability. Second, data were collected through an online survey, which likely overrepresents young, highly educated individuals with stronger scientific literacy. This sampling bias may have amplified the prominence of scientific worldviews while underrepresenting supernatural or religious perspectives. Future studies should aim to include a larger proportion of participants who endorse supernatural or religious beliefs to improve the generalizability of the findings. Third, the cross-sectional nature of the data introduces ambiguity regarding the causal direction of the observed relationships. Future longitudinal or experimental designs are needed to establish causality. Finally, the findings of this study offer empirical evidence for the conflict thesis regarding religious and scientific beliefs in social issues, highlighting the need to approach religiosity and science with greater nuance than in previous studies. However, given the specific population and context of this study, caution should be exercised when generalizing the findings beyond these bounds.

## Conclusion

Although various factors contribute to cancer prevention, social-cognitive resources have received comparatively less scholarly attention. This study demonstrates that supernatural and scientific beliefs serve as significant predictors of cancer control beliefs and clarifies the pathways through which these worldviews influence cancer prevention behaviors. These findings highlight key intervention strategies: reducing fatalistic narratives about cancer while promoting scientific attitudes and cancer controllability in prevention efforts. Compared to traditional approaches, such as cancer knowledge education or fear appeals, interventions centered on worldview modification may prove more essential. Young adults typically exhibit low perceptions of cancer risk alongside high health expectations, limiting the impact of conventional fear appeal-based methods. Instead, cultivating accurate cancer control beliefs can fundamentally support long-term individual prevention practices. Naturally, more empirical studies are required to substantiate these conclusions.

## Data Availability

The raw data supporting the conclusions of this article will be made available by the authors, without undue reservation.

## Footnotes

1 According to the World Health Organization, prevention offers the most cost-effective long-term strategy for the control of cancer, and 30–50% of all cancer cases are preventable. Common and fundamental risk factors include tobacco use, alcohol consumption, physical inactivity, diet, and environmental exposures (https://www.who.int/activities/preventing-cancer).

2
https://www.credamo.com

3 According to the United Nations Educational, Scientific and Cultural Organization (UNESCO) (2013), individuals aged 10–24 years are classified as adolescents and young people (84). However, other scholars argue that young adulthood should be determined by subjective markers of adulthood rather than biological transitions (such as the ability to bear children) and therefore extend the age range to include people aged 20–39 or even 20–44 years (85). In health-related research, the age range of 20–45 years has also been adopted as the baseline population for studying young adults (86, 87). In light of these varying definitions, this study defined young adults as participants aged 18 to 40 years.

4 The minimum sample size was determined using two established approaches. First, by applying the formula for estimating a proportion in a finite population, *N* = *Z* × *p*(1-*p*)/*e^2^*, we set *Z* at 1.96 (for a 95% confidence interval), *p* at 0.5 (to ensure a conservative, maximum sample size), and the margin of error (e) at 5%. This calculation resulted in a minimum sample size of 384 participants. Second, in line with the recommended practice in structural equation modeling, where the sample size should be at least 10 times the number of measured variables, and with fewer than 40 items in our instrument, the sample size needed to exceed 400. To ensure robustness, we distributed 1,002 questionnaires. After quality screening, 938 valid responses were retained, a number that fulfills both methodological requirements and supports the validity of our subsequent analyses.
