# Correction: How supernatural and scientific beliefs influence individual cancer prevention behaviors: an empirical study of young Chinese adults

**DOI:** 10.3389/fpubh.2025.1764647

**Published:** 2026-01-20

**Authors:** Qinliang Liu, Hepeng Jia

**Affiliations:** School of Communication, Soochow University, Suzhou, China

**Keywords:** cancer prevention, young adults, scientific attitudes, supernatural/religion, health control beliefs

The images in “Figure 1. The framework of the research questions” and “Figure 2. The path coefficients of the structural model” were in the wrong order. in the PDF and HTML version of this paper. The positions of Image 1 and Image 2 should be swapped. The correct order should be as follows:

**Figure 1:**
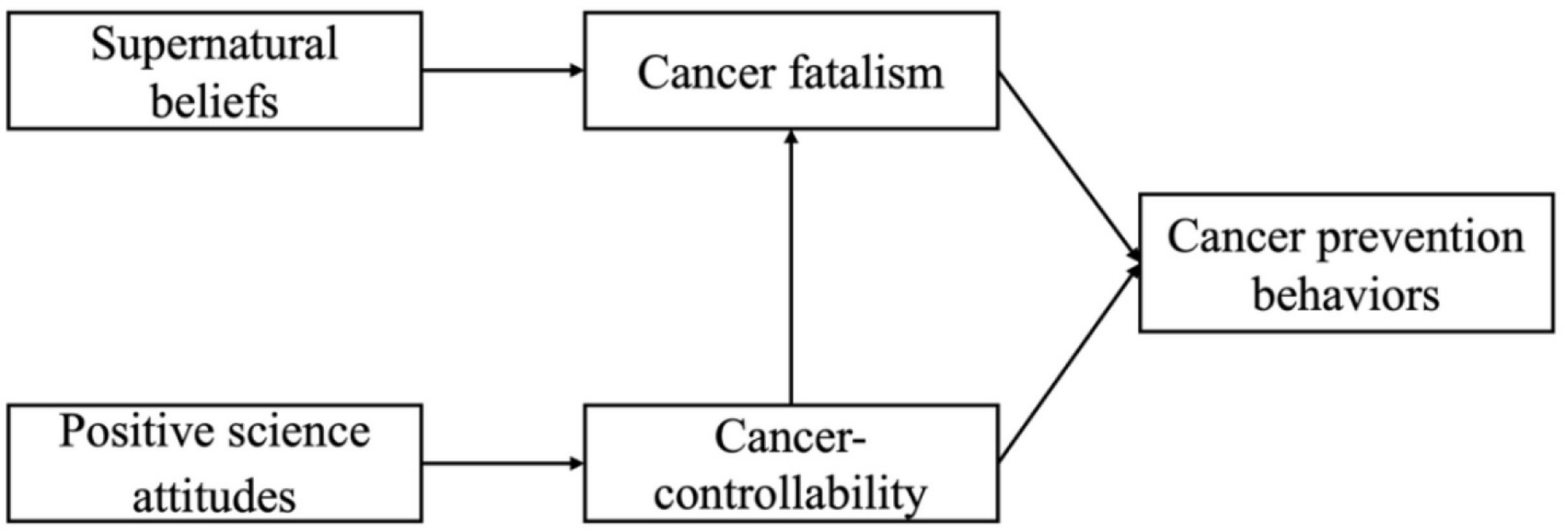
The framework of the research questions

**Figure 2:**
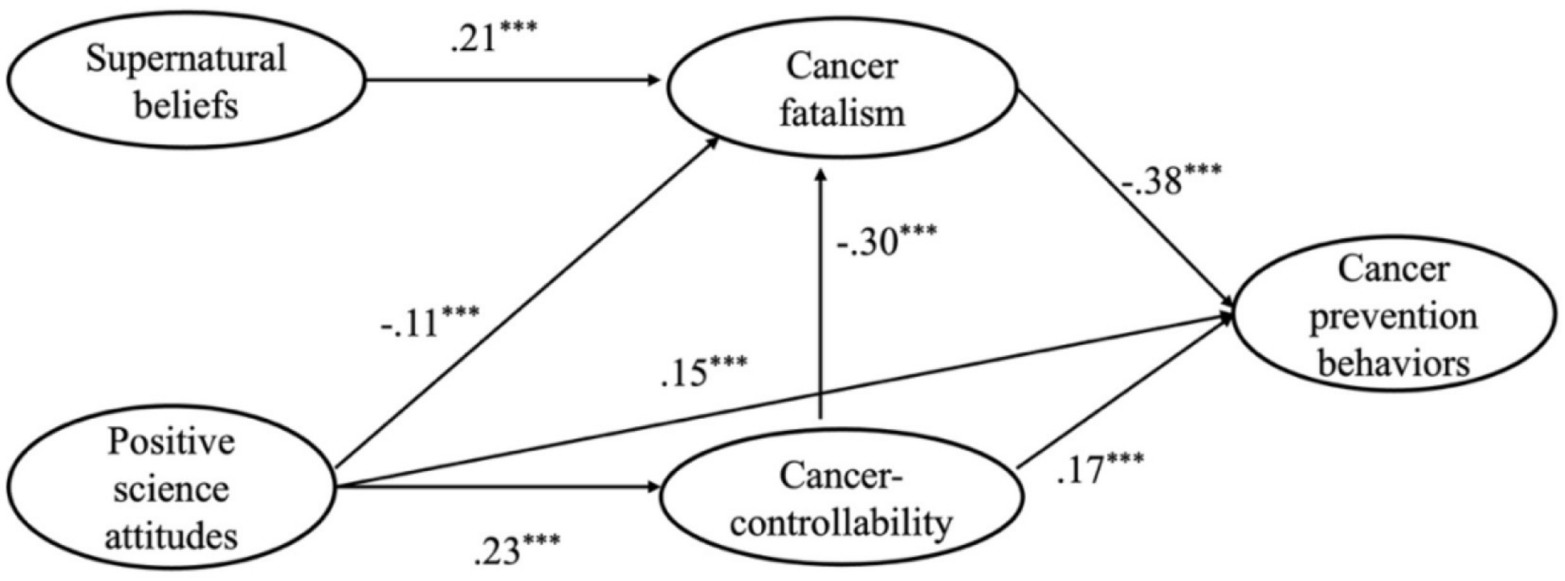
The path coefficient of the structural model.

The order has now been corrected.

The original version of this article has been updated.

